# Applications of single-cell RNA sequencing in atopic dermatitis and psoriasis

**DOI:** 10.3389/fimmu.2022.1038744

**Published:** 2022-11-25

**Authors:** Dengmei Xia, Yiyi Wang, Yue Xiao, Wei Li

**Affiliations:** ^1^ Department of Dermatology, Rare Diseases Center, West China Hospital, Sichuan University, Chengdu, Sichuan, China; ^2^ Department of Dermatology, The Affiliated Hospital of Southwest Medical University, Luzhou, Sichuan, China; ^3^ Institutes for Systems Genetics, Frontiers Science Center for Disease-related Molecular Network, National Clinical Research Center for Geriatrics, West China Hospital, Sichuan University, Chengdu, Sichuan, China

**Keywords:** single-cell RNA sequencing, inflammation, atopic dermatitis, psoriasis, transcriptomics

## Abstract

Single-cell RNA sequencing (scRNA-seq) is a novel technology that characterizes molecular heterogeneity at the single-cell level. With the development of more automated, sensitive, and cost-effective single-cell isolation methods, the sensitivity and efficiency of scRNA-seq have improved. Technological advances in single-cell analysis provide a deeper understanding of the biological diversity of cells present in tissues, including inflamed skin. New subsets of cells have been discovered among common inflammatory skin diseases, such as atopic dermatitis (AD) and psoriasis. ScRNA-seq technology has also been used to analyze immune cell distribution and cell-cell communication, shedding new light on the complex interplay of components involved in disease responses. Moreover, scRNA-seq may be a promising tool in precision medicine because of its ability to define cell subsets with potential treatment targets and to characterize cell-specific responses to drugs or other stimuli. In this review, we briefly summarize the progress in the development of scRNA-seq technologies and discuss the latest scRNA-seq-related findings and future trends in AD and psoriasis. We also discuss the limitations and technical problems associated with current scRNA-seq technology.

## Introduction

Inflammatory skin diseases are induced by skin barrier disorders and dysregulation of innate and adaptive immunity. Atopic dermatitis (AD) and psoriasis are two of the most common chronic inflammatory skin diseases (1, 2). The Global Burden of Disease study showed that AD is the 15th most common non-fatal disease and the skin disorder with the highest disease burden, with a prevalence of 15% to 20% among children and up to 10% in adults (3). Approximately 125 million people worldwide are estimated to have psoriasis (4). Although genetic, immune dysregulation, and environmental factors play important roles in the pathogenesis of AD and psoriasis (1, 2), the detailed mechanisms of AD and psoriasis remain unclear. In recent years, specific immune component targeted therapies have been reported in AD and psoriasis, with substantially positive effects. However, some patients do not respond to these treatments, have a secondary failure, or relapse after drug withdrawal; thus, the underlying mechanisms regarding the treatment of these diseases remain unclear.

Bulk RNA sequencing is an indispensable tool for analyzing transcriptional variations, which determine the average gene expression among pooled populations of cells and reported as single data. However, tissues consist of multiple cell types in various states; hence, the results of such a technique can be misleading. Newly developed single-cell RNA sequencing (scRNA-seq) technologies facilitate the analysis of transcriptional activity at the single-cell level (5). scRNA-seq facilitates the assessment of cellular heterogeneity, identification of new or rare cell populations, and clarification of cellular transition states at a high resolution. In addition, Thus, organ- or tissue-specific transcriptomic characteristics of keratinocytes (KCs), fibroblasts, endothelial cells, and immune cells hosted within or infiltrated after inflammation can be assessed to elucidate the function of cell heterogeneity in AD and psoriasis.

## A brief introduction to single-cell RNA sequencing

scRNA-seq is a powerful tool for providing precision and detail to individual cells (6). The workflow of scRNA-seq usually includes sample preparation, single-cell capture, reverse transcription of full-length mRNA, cDNA amplification, preparation of a sequencing library, high-throughput sequencing, and bioinformatics analyses. Single-cell isolation and amplification of cDNA are the main steps in various single-cell sequencing strategies.

Methods of traditional single-cell isolation have eventually developed after many optimizations. Micromanipulation is a classic technique for manually capturing under a microscope (7, 8). It can accurately select single cells under microscopic observation and is suitable for analyzing a limited number of cells (9). However, this method is time-consuming, has low throughput, and may cause cellular injury due to mechanical shearing (10). Laser capture microdissection (LCM) is another approach for obtaining single cells from solid tissue. In this technique, a laser beam is used to capture the cells of interest from the tissue specimen quickly and accurately, attaching these cells to a thin and transparent film (11). Here, the spatial positional information of the target cells is retained (12); however, in addition to being laborious and inconvenient, there is a risk of destroying the integrity of cells and damaging cellular RNA (13), which could impact subsequent analyses. Fluorescence-activated cell sorting (FACS) is a specialized type of flow cytometry that sorts large numbers of cells based on cell surface markers and physicochemical properties and completes quantitative analyses quickly (14). Time consumption and low throughput limit the use of these traditional technologies. To circumvent this, scRNA-seq technology platforms have been rapidly developed based on the application of microfluidic and single-cell identification technologies.

Novel single-cell capture methods based on microfluidics include integrated fluidic circuits (IFCs), droplets, microwells, traps, and the SlipChip (15). Popular platforms have recently enabled droplet-based scRNA-seq, which sorts cells into aqueous compartments in a lipid suspension (16). Using this system, the cell capture rate of a single sample can be as high as 65%, and 80,000 cells can be simultaneously isolated and amplified in minutes (17). Split pool ligation-based transcriptome sequencing (SPLiT-Seq) uses combinatorial barcodes to label individual cells (18), which is expected to decrease operational costs and does not involve microfluidic devices. The development of these technologies has led to the widespread use of scRNA-seq. The advantages and disadvantages of these single-cell isolation methods are summarized in **Table 1**.

**Table 1:** Comparison of the advantages and disadvantages of single-cell isolation methods.

Techniques	Automation level	Impact on cell integrity	Advantages	Disadvantages
Micromanipulation (8–10)	Manual	Gentle	Precise capturing of single cells under direct visualization. Low cost.	Time-consuming and low throughout.
Laser capture microdissection (LCM) (11–13)	Manual	Often impairing	Isolation of single cells from solid samples, not need the preparation of cell suspensions.	Time-consuming, low throughout. and influence subsequent amplification.
Fluorescence-activated cell sorting (FACS) (14)	Automatic	Often impairing	Suitable for sorting different types of cells. High hroughout.	Hard to detect cellular characteristics expressed at a low level and differentiate with similar marker expressions.
Integrated fluidic circuits- based microfluidics (15)	Automatic	Few	Capture and process 800 small or medium-size single cells simultaneously.	High cost.
Microdroplet-based microfluidics (16, 17)	Automatic	Few	Isolation and amplification of 80,000 cells imultaneously in minutes.	A risk of blockage.
Microwell-based microfluidics (15)	Automatic	Few	Single cells settled by gravity and microwells replaced droplets. Simultaneously capture approximately 10,000 single cells.	May not provide adequate space for the proliferation and movement of the cells.
Split pool ligation-based transcriptome sequencing (SPLiT-Seq) (18)	Automatic	/	Independent to microfluidic devices and low cell requirement of samples.	/

/: Not mentioned.

Single-cell bioinformatics analysis, typically involves fundamental analytical procedures such as quality control, normalization, dimensionality reduction, differential expression gene analysis, visualization, clustering, and cell type annotation. Thus, this analysis can obtain the basic single-cell landscape of specific disease. Some advanced analyses, such as trajectory and cell-cell communications, can help us further capture specific disease-related cells, genes, functional pathways, and cell-cell interactions. scRNA-seq technology has been applied in skin cancer and autoimmune skin diseases. For example, ferroptosis-related genes and resident memory CD8+ T cells in regional lymph nodes have been identified to predict the prognosis of melanoma using scRNA-seq analyses (19, 20). Moreover, patterns of dedifferentiation in melanoma are predictive of the response to immune checkpoint inhibitor therapy (21). Type 1 cytokine signaling plays a central role in vitiligo, and Treg cells inhibit disease progression in non-lesional skin (22). A unique cluster of CXCL13+ T cells identified *via* scRNA-seq appears to promote B- cell responses within the inflamed skin of patients with systemic sclerosis (23). These data provide critical insights into the pathogenesis of melanoma, vitiligo, and systemic sclerosis, respectively.

## Applications of single-cell RNA sequencing in inflammatory skin diseases

### Atopic dermatitis

Atopic dermatitis (AD) is one of the most common inflammatory skin diseases. The prevalence of AD has been reported up to 20% among children and 10% among adults (24), and the causes are complex. Genetic susceptibility, a dysfunctional epidermal barrier, skin microbiome abnormalities, and type-2 immune dysregulation predominantly play a role in the pathogenesis of AD (2). The endotype is the molecular mechanism underlying the disease phenotypes (25, 26). Aside from the presence of IgE that can distinguish between intrinsic and extrinsic AD, AD is characterized by a highly diverse endotype repertoire, including the dysregulation of Th1/Th2/Th17/Th22 cells and impaired epidermal barrier integrity (27).

In addition to the unclear specific pathogenic mechanisms of AD, the treatment options for this disease vary. Targeted therapies for specific AD endotypes, such as those directed against Th2/Tc2, and Th17 cells and general anti-inflammatory agents, have been proved by the FDA or are currently in different phases of clinical trials (28). Additionally, addressing the unsatisfactory efficacy of current therapies and identifying biomarkers that will improve therapy selection for biological agents and small-molecule drugs should be the focus for AD.

RNA sequencing and gene microarray analysis of skin biopsy specimens have provided insights into AD pathogenesis (29, 30). scRNA-seq was performed on lesional and non-lesional samples from patients with AD and skin from healthy individuals. It was found that *COL6A5^+^COL18A1 ^+^* fibroblast, which express the cytokines CCL2 and CCL19, were a novel cell subpopulation unique to AD lesional skin. A dendritic cell population that expresses the CCL19 receptor CCR7 is also unique to AD lesions (31). Prx1+ fibroblasts overexpressing the eosinophilic chemokine CCL11 may also contribute to the pathogenesis of AD by dysregulating *IKKβ/NF-κB* signaling; hence, targeting CCL11 upregulation in Prx1+ fibroblasts may be a way to treat AD-like skin diseases (32). Whether these subpopulations of fibroblasts recruit T cells and other inflammatory cells into the local lesions or play a role in the initiation, maintenance, and regression phases needs further research.

Meanwhile, myeloid dendritic cells (DCs), including inflammatory dendritic epidermal cells, form the most expanded immune cell population in AD lesions (33). It has been demonstrated that unique inflammatory fibroblasts may interact with immune cells, such as DCs expressing CCR7, to regulate type 2 inflammation. Moreover, innate lymphoid cells (ILC), especially ILC2, have been implicated in AD pathogenesis. ILC2s are activated by various tissue-derived factors and exhibit different functions in both the steady- state and inflammation. The number of ILC2s in lesional skin biopsies from patients with AD was significantly higher than in healthy individuals (34, 35). Skin ILC2s were further sub-classified into skin-resident and circulating ILC2s through scRNA-seq in a transgenic mouse line expressing skin-specific IL-33 expression. Here, these transgenic mice showed ILC2- dependent atopic dermatitis-like skin inflammation (36). Patients with AD have a high percentage of ILC2s in their peripheral blood that respond better to IL-4/13 inhibitors, such as dupilumab (37). Furthermore, ILC2s in lesional AD skin have been shown to be biologically heterogeneous and are involved in the IL-33 signaling pathway. ILC2s are flexible and co-express typical genes, either type 2 or type 3/17 immunity markers, within individual cells (38). It is well known that type3/17 immunity is associated with the development of psoriasis. However, whether the plasticity of ILC2s is responsible for psoriasis-related dermatitis remains unclear.

AD typically starts in infancy or early childhood, showing spontaneous regression after puberty in a subset of patients while waxing and waning for life in many others. However, the factors that modify the natural course of spontaneous remission remain to be elucidated. The overall cell composition of patients with spontaneously healed AD was comparable to that of healthy individuals. Compared to healthy controls, melanocytes exhibit many differentially expressed genes in all cell types in spontaneously healed atopic dermatitis. Specifically, the expression of the potential anti-inflammatory, maker PLA2G7 (Lipoprotein-associated phospholipase A2 or “platelet-activating factor acetylhydrolase) is increased. Regulatory markers are also upregulated in conventional T-cells (39). Moreover, skin-resident memory T cells showed the greatest transcriptional dysregulation in AD (40), which may be responsible for the recurrence of the disease. KCs also play an important role in the pathogenesis of AD. Epidermal proliferation and chemokines (CCL2 and CCL27) were significantly upregulated in the KCs of lesional AD. Such KCs were found to be enriched during epidermis development and immune responses (31).

Based on previous studies, innate immune cells (ILC2s and DCs), fibroblasts, and KCs, as well as their interplay and interactions, play a role in AD development. Melanocytes and skin-resident memory T cells may contribute to the specific regulatory microenvironment in the spontaneous remission and recurrence of AD, respectively.

### Psoriasis

Psoriasis is also a common, chronic inflammatory skin disease, and its incidence in ethnic groups and countries is significantly different (41). The pathogenesis of psoriasis is complex and multifactorial, involving genetic, immune, and environmental factors. The IL-23/Th17 pathway is thought to be the predominant pathway governing the progression and development of psoriasis (42). Biologics that target IL-17/IL-17 receptor and IL-23 have shown significant clinical efficacy in patients with psoriasis (43, 44). Cutaneous type 17 T-cells showed markedly different g transcriptome profiles depending on various cytokines, including IL-17A, IL-17F, and IL-10 (45). CD8+ T cells are increased in abundance within psoriatic lesions (46) and are found to produce inflammatory cytokines, such as IL-17, at sites of the active phase of psoriasis (47). However, CD8+ T cells are phenotypically heterogeneous and have distinct functional properties with cytotoxic and cytokine-producing features (48). Two pathogenic cytotoxic type 17 T-cell (Tc17) subsets of CD8+ T cells were identified in psoriatic skin from lesional skin biopsies of 11 patients with psoriasis and five healthy control individuals *via* single-cell transcriptomics. CXCL13- expressing Tc17 cells appear to be specific to psoriatic lesions and are associated with disease severity (49). Up to 30% patients with psoriasis may develop psoriatic arthritis (PsA), presenting with peripheral arthritis, enthesitis, and (or) dactylitis (50). The expansion of memory CD8+ T cells in the joints of PsA patients was significantly higher than that in their peripheral blood. CD8+ T cells have also been previously reported in the synovial fluid of PsA patients (51). single-cell sequencing showed that in the synovial fluid, CD8+T cells that express CXCR3, a tissue-homing receptor, are increased in abundance and that the expression of its ligands (CXCL9 and CXCL10) were elevated, providing molecular insight into the cellular immune mechanism of PsA (52). Compared to healthy, patients with psoriasis are characterized by T_reg_ expansion and CD8+ T cell exhaustion. Moreover, differentially expressed genes in skin-resident memory T cells have been recently reported to discriminate psoriasis vulgaris from AD. Other T cell subsets, such as dysfunctional T cells that regulate and express NR4A1, are also involved in psoriasis (53).

In addition to adaptive immunity, innate immunity plays an important role in the pathogenesis of psoriasis. Cutaneous antigen-presenting cells (APCs) are divided into three groups: Langerhans cells in the epidermis, classical dendritic cell type 1(cDC1) and cDC2 in the dermis, and macrophages (54). A new subset of inflammatory DCs expressing CD5-CD163+CD14+ (DC3) was identified in human blood (55). CD14+ DC3 cells expressing genes related to IL-17 and neutrophil activation signaling were enriched in psoriatic lesions, which were considered potential promoters of inflammation in psoriasis. Higher proportions of macrophage-expressing genes related to inflammatory chemokines and cytokines (CXCL8 and CXCL2) were found in psoriatic lesions compared to non-lesional skin (56). ILC3s have been reported in human and mouse psoriatic lesions (57, 58). The response to therapeutic compounds has decreased the number of ILC3 cells (59). Fate mapping analysis suggested that ILC3-like cells may arise from quiescent-like cells and ILC2s, highlighting the flexibility of skin ILC responses and driving the pathological remodeling process (60).

Even though immune cell infiltration plays a fundamental role in cutaneous inflammation, KCs can also influence the inflammatory microenvironment (61). scRNA-seq analysis showed that aberrant inflammatory transcription of A20 in KCs in psoriasis is related to the IL-17 and tumor necrosis factor-α(TNF-α) signaling pathways (62), suggesting a potential targeted therapy. Hence, Th17 cells, CD8+ T cells, DC3 cells, macrophages, ILCs and KCs play an important role in the development of psoriasis. And Tc17 cells, a subtype of CD8+ T cells, are associated with disease activity.

## Discussion

Since 2009, the first conceptual and technical breakthrough of the single-cell RNA sequencing method was made by Tang et al. (8). An increasing number of improved scRNA-seq technologies were developed to introduce essential modifications and improvements in sample collection, single-cell capture, barcoded reverse transcription, cDNA amplification, library preparation, sequencing and streamlined bioinformatics analysis. Freshly tissues, high cost of per sample and scRNA-seq data analysis still remain challenge. Subsequent studies are expected to explore fixed tissue sample and to downregulate costs. And automatic sc-RNA-seq data analysis pipelines and visualization platforms are expected to be available in the future.

Most of studies related in this review prepare the libraries and sequence depending on 10X Genomics platform. These findings highlight KCs, fibroblasts, and different types of immune cells in mechanisms for coping with the different stages of AD and psoriasis. scRNA-seq offers a novel method to identify the receptors, ligands, and cytokines expressed in each cell type to further highlight intercellular communication in the skin microenvironment. However, the detailed mechanisms driving the pathogenicity of these cells and the relationship between them require further study. And the clinical characteristics of AD and psoriasis varied. Therefore, more specific studies are necessary to elucidate the characteristics of AD and psoriasis based on the differences in the stages and subtypes of these diseases. Patients with clinical characteristics of both AD and psoriasis have been described as having psoriasis dermatitis, typically found in children (63). In addition to the co-existence of both AD and psoriasis, disease co-occurrence but alternating flare-ups or co-occurrence at different life stages may also be observed (64). The overlap condition not only presented in children but also in adult patients. With the increasing application of biologics agents, the psoriasiform reaction during dupilumab therapy has been reported in AD, and an eczematous reaction to anti-interleukin (IL)-17 treatment has been reported in psoriasis (65, 66). However, the pathophysiology should be further clarified. Moreover, it will be beneficial to analyze cell lineage trajectories and patient-specific cell heterogeneity using scRNA-seq data.

Analyzing the non-coding RNAs (ncRNAs) with scRNA-seq and combining proteomics with epigenomics will get closer to a ture global examination of single cell. In addition, the analysis of minimally invasive or non-invasive samples, such as blood or urine specimens, may also hold promise in diagnosis process and treatment response prediction. Considering the rapid development of sequencing methods, scRNA-seq can be expected to enter the clinics soon and facilitate personalized therapeutic decisions for patients with inflammatory skin diseases.

## Limitations of single-cell RNA sequencing

Although scRNA-seq can identify cell type-specific transcriptional regulation and cellular heterogeneity, its limitations can be challenged. Firstly, high-throughput single-cell analysis requires cell dissociation, quality control, and are largely tend to examine freshly isolated cells. More research will use such technology to explore cryopreserved and fixed tissue samples. And the preparation of single-cell suspensions destroys spatial information of tissues. In addition, researchers have mostly focused on protein-coding RNA. An increasing number of studies have indicated that ncRNAs have important roles in cell function and specialization (67–69). Even though it has been neglected in previous scRNA-seq studies, nanopore sequencing and Smart-seq-total technology may also address current gaps in the technology (70, 71). Bioinformatics analysis of DEGs in scRNA-seq strongly depends on the cell count in each identified cluster, whether the transcriptional changes obtained *via* scRNA-seq are specific biological findings, or a biased subset clustering data that is prone to misinterpretation (72, 73). The development of scRNA-seq technology has raised a wide range of computational and analytical challenges. Even though several methods have now been designed to efficiently perform upstream (quality control and normalization) and downstream (cell-, gene- and pathway-level) analyses of scRNA-seq data (74), there are limited guidelines on how to define quality control standards, remove technical artifacts, and interpret results. Deep-learning based methods, such as machine learning, may also provide more benefits than traditional statistical models in dealing with high-dimensional data.

## Conclusions

**Figure 1:**
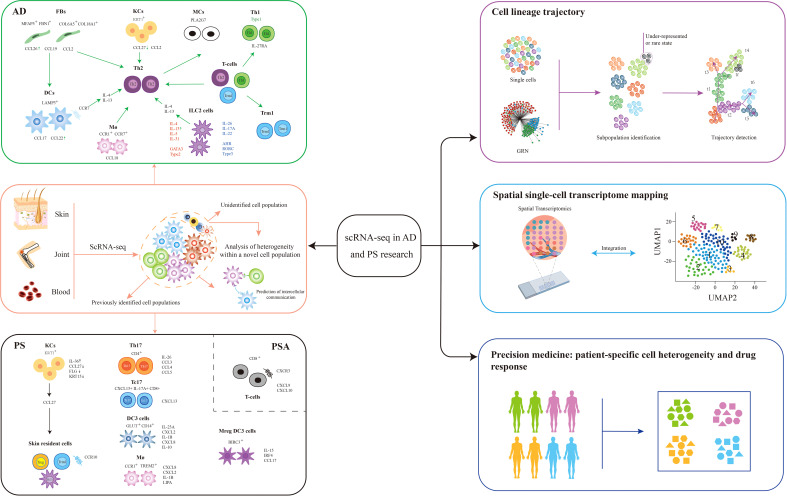
Application of scRNA-seq in AD and PS. Single-cell RNA sequencing (scRNA-seq) is especially useful in the detection of rare cell populations, identifying cell-to-cell interactions, reconstructing skin cell trajectories, spatial transcriptomic mapping of skin (75), and developing more precision medicine tools for the better prediction of patient-specific drug responses. The future trends involved cell-to-cell communication, skin cell trajectories, spatial transcriptomic mapping of skin, and precision medicines in these diseases. AD, atopic dermatitis; PS, psoriasis; FBs, fibroblasts; KCs, keratinocytes; MCs, melanocytes; Th1, T helper 1; Th17, T helper 17; Tc17, expressing IL-17 cytotoxic CD+8 T cell; Trm, skin-resident memory T cell; Treg, regulatory T cell; DC, dendritic cell; Mreg DC, mature dendritic cell enriched in immunoregulatory molecules; ILC, innate lymphoid cell; Mø, macrophage; CCL, CC chemokine ligand; CXCL, C-X-C motif ligand; GRN, gene regulatory network; t-SNE, t-distributed stochastic neighbor embedding.

In recent years, transcriptomics has made a great leap from bulk RNA-seq, which measures the average gene expression, to analyzing gene expression data in individual cells. This mini-review summarizes and discusses the applications of scRNA-seq in AD and psoriasis (**Figure 1**). Single-cell RNA sequencing has provided new insights into inflammatory skin disease heterogeneity, revealed complex interactions between cell types, and allowed a more comprehensive understanding of inflammatory skin disease initiation, progression, and regression. New insights are crucial for developing targeted and innovative therapeutic strategies, to advance precision medicine for inflammatory skin diseases. Although some limitations remain, scRNA-seq will pave the way for personalized medicine once solving the challenges.

## Author contributions

DX wrote the manuscript. YW, YX, and WL contributed to the revision of the manuscript. All authors approved the final submitted version.

## Funding

1·3·5 project for disciplines of excellence, West China Hospital, Sichuan University.

## Conflict of interest

The authors declare that the research was conducted in the absence of any commercial or financial relationships that could be construed as a potential conflict of interest.

## Publisher’s note

All claims expressed in this article are solely those of the authors and do not necessarily represent those of their affiliated organizations, or those of the publisher, the editors and the reviewers. Any product that may be evaluated in this article, or claim that may be made by its manufacturer, is not guaranteed or endorsed by the publisher.
